# Ultra-high resolution photon-counting detector coronary CT angiography: diagnostic accuracy in patients with high Agatston scores

**DOI:** 10.1007/s00330-025-12158-z

**Published:** 2025-11-20

**Authors:** Tristan T. Demmert, Konstantin Klambauer, Lukas J. Moser, Jonathan Michel, Markus Kasel, Robert Manka, Victor Mergen, Thomas Flohr, Matthias Eberhard, Hatem Alkadhi

**Affiliations:** 1 Diagnostic and Interventional Radiology, University Hospital Zurich, University of Zurich, Zurich, Switzerland; 2 Department of Cardiology, University Hospital Zurich, University of Zurich, Zurich, Switzerland; 3 Department of Radiology and Nuclear Medicine, Maastricht University Medical Centre+, Maastricht, The Netherlands

**Keywords:** Calcification, Agatston score, Coronary, ICA, CCTA

## Abstract

**Objectives:**

Severe coronary artery calcification leads to blooming artifacts in conventional coronary CT angiography (CCTA), which may reduce diagnostic accuracy. Ultra-high resolution (UHR) photon-counting detector CT (PCD-CT) reduces calcium blooming. This study aimed to evaluate the diagnostic performance of UHR CCTA with PCD-CT for detecting coronary stenoses in patients with a high coronary calcium burden compared to invasive coronary angiography (ICA).

**Materials and methods:**

In this IRB-approved single-center study, patients with Agatston scores > 600 were included, who underwent ICA and coronary UHR PCD-CT angiography within 2 months. 94% of patients underwent CT prior to transcatheter aortic valve replacement. Coronary arteries were graded for diameter stenosis severity (> 50, > 70%) by two blinded, independent readers. Accuracy, sensitivity, and specificity of CCTA were calculated using ICA as the reference standard. Subgroup analyses were performed across Agatston score groups (600–999, 1000–1999, 2000–2999, and > 3000).

**Results:**

62 patients (mean age, 76 ± 9 years, 16 women) were included. The median Agatston score was 2423 (IQR: 1085–3340). Across all Agatston categories, CCTA demonstrated high diagnostic accuracy and strong agreement with ICA (Cohen’s kappa 0.71–1.00). In patients with Agatston scores > 3000 (*n* = 18), the diagnostic performance of coronary UHR PCD-CT angiography for detecting > 50% (sensitivity 92–100%, specificity 83–100%) and > 70% diameter stenosis (sensitivity 88–100%, specificity 89–100%) remained high for both readers. There was no correlation between the Agatston score and diagnostic error (*p* > 0.05).

**Conclusion:**

UHR CCTA with PCD-CT provides high diagnostic performance even in patients with extensive coronary calcifications.

**Key Points:**

***Question*** *In patients with severe coronary calcification, conventional coronary CT angiography often suffers from blooming artifacts, reducing accuracy and causing unnecessary invasive procedures.*

***Findings*** *Ultra-high resolution coronary angiography with photon-counting detector CT showed high sensitivity and specificity for detecting stenoses, even at Agatston scores > 3000.*

***Clinical relevance*** *Ultra-high resolution coronary CT angiography enables accurate noninvasive detection of stenoses in heavily calcified vessels, potentially reducing invasive coronary angiography.*

**Graphical Abstract:**

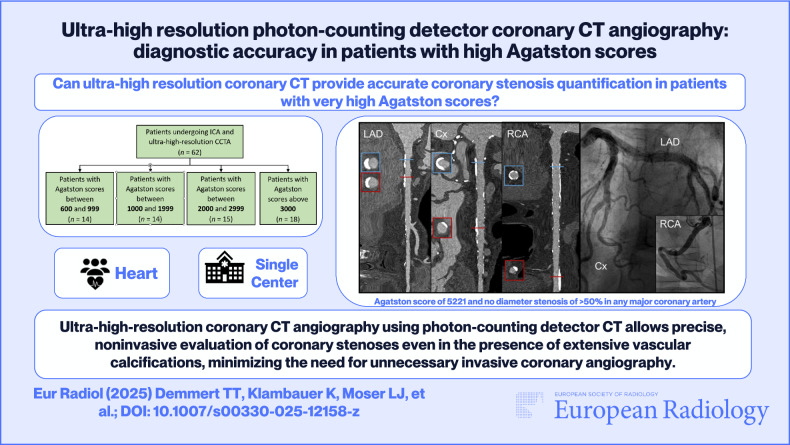

## Introduction

Coronary computed tomography angiography (CCTA) represents the first-line imaging modality in patients with low and intermediate pre-test probability of coronary artery disease (CAD) [1, 2]. In patients with a higher probability, current guidelines recommend functional tests to assess for myocardial ischemia [3]. One of the reasons why CCTA is not the primary modality in the latter patient group is its reduced accuracy in the presence of severe coronary calcifications. Such calcifications may cause blooming artifacts [4] that obscure the perfused coronary vessel lumen, potentially leading to stenosis overestimation, thereby reducing the specificity of the modality [5]. False positive coronary stenoses according to CCTA may lead to unnecessary downstream invasive coronary angiography (ICA) [6]. Prior studies using conventional energy-integrated detector CT (EID-CT) observed a decline in diagnostic performance in patients with Agatston scores ≥ 600 [7] or ≥ 1000 [8].

The recently introduced photon-counting detector CT (PCD-CT) enables ultra-high resolution (UHR) CCTA with a maximum in-plane resolution of 0.11 mm and a maximal through-plane resolution of 0.16 mm [9], thereby reducing blooming artifacts from coronary calcifications. Early studies suggested that UHR PCD-CCTA could potentially enable accurate assessment of coronary stenoses also in patients with higher Agatston scores [10, 11]; however, the number of included patients with high calcium scores in these studies and the literature in general on this topic remains sparse.

The purpose of this study was to evaluate the diagnostic performance of UHR PCD-CCTA for detecting coronary stenoses in patients with a high coronary calcium burden compared to ICA.

## Materials and methods

### Study design and patient population

This retrospective single-center study was conducted in accordance with the Declaration of Helsinki, was approved by the institutional review board and ethics committee and followed the STARD guidelines [12]. Consecutive patients referred for UHR PCD-CCTA between April 2022 and April 2025 were screened for study inclusion. The vast majority of patients (94%) were referred for pre-procedural assessment of transcatheter aortic valve replacement (TAVR). Eligible patients fulfilled the following criteria: (1) age ≥ 18 years; (2) Agatston scores > 600; (3) ICA performed within 2 months of CCTA with no coronary intervention in between; (4) availability of CCTA with PCD-CT scanned in the UHR mode. Exclusion criteria included foregoing coronary revascularization, including both coronary artery bypass grafting and coronary stenting.

### CT data acquisition and reconstruction

All examinations were performed using a first-generation dual-source photon-counting CT scanner (NAEOTOM Alpha, Siemens Healthineers AG) in the UHR mode. Image data was acquired with a tube voltage of 120 or 140 kVp and a detector collimation of 120 × 0.2 mm. A non-contrast scan for calcium scoring preceded contrast-enhanced UHR CCTA. The median volume CT dose index (CTDI_vol_) of CCTA was 41.2 mGy (IQR 25–53 mGy) and the median dose length product was 672 mGy·cm (IQR 341–872mGy·cm). The average heart rate during data acquisition was 73 ± 15 bpm. A triphasic injection protocol was used with 50–80 mL of iodinated contrast media (iopromide, Ultravist 300 mgI/mL, Bayer Healthcare), tailored to patient body mass index (BMI), followed by a saline flush. Bolus tracking was applied with a region of interest placed in the ascending aorta. Images were reconstructed using a 512 × 512 matrix, a field of view of 200 × 200 mm, and a sharp vascular kernel (Bv60) at 0.2 mm slice thickness (increment 0.2 mm), as previously recommended [9]. Sublingual nitrates were administered prior to CT, unless contraindicated.

### Invasive coronary angiography

ICA served as the reference standard. For this study, the original clinical ICA reports, as issued by board-certified interventional cardiologists, were used. No additional retrospective re-reading of the angiograms was performed. ICA was performed according to institutional standards via radial or femoral access using standard selective coronary catheterization techniques [13]. Iodinated contrast media (Iopromide, Ultravist 300 mgI/mL, Bayer Healthcare) was used for vessel opacification. All angiograms were acquired in multiple orthogonal projections to ensure comprehensive coronary visualization.

### Image interpretation

The Agatston score was determined from non-contrast CT using a standard clinical software package (syngo.via, Siemens). The software automatically calculated the score following the method originally described by Agatston et al [14]. Each result was visually reviewed by an experienced reader not involved in further readouts.

Two board-certified radiologists (H.A. and M.E., each with > 10 years of experience in cardiovascular CT) independently reviewed the CCTA datasets, blinded to the ICA findings. Coronary arteries were segmented according to the AHA 17-segment model. The left main coronary artery (LM), left anterior descending artery (LAD), left circumflex artery (Cx), and right coronary artery (RCA) were assessed. Diameter stenosis severity was visually categorized, adapted to the CAD RADS 2.0 classification [15]: < 50%, 50–70%, and > 70%. Analyses were performed on an intention-to-diagnose basis. Accordingly, all patients fulfilling the inclusion criteria were evaluated, and non-diagnostic coronary segments were conservatively classified as positive for stenosis. This approach avoids overestimation of specificity and reflects routine clinical decision-making. In ICA, the stenosis severity was assessed by two interventional cardiologists, using the same categorization as for CCTA.

### Statistical analysis

Descriptive statistics were reported as mean ± standard deviation (SD) or median with interquartile range (IQR), depending on distribution (Shapiro–Wilk test). CCTA diagnostic performance was assessed at the patient- and vessel-level using ICA as the reference standard. Sensitivity, specificity, and accuracy for detecting > 50% and > 70% stenoses were calculated together with two-sided 95% confidence intervals (exact binomial). Positive and negative predictive values are reported in the supplement as the retrospectively assembled cohort was intentionally enriched for disease (pre-TAVR workup), rendering positive and negative predictive values non-generalizable; we therefore focused on prevalence-invariant metrics. For the analysis of baseline characteristics, categorical variables were compared between groups using Fisher’s exact test, and continuous variables were assessed using Welch’s *t*-test or the Mann–Whitney U test, depending on distribution (Shapiro–Wilk test). For vessel-level analysis of discrepancy versus concordance between ICA and CCTA, the same testing strategy was applied.

To assess whether diagnostic performance declined at higher coronary calcium burden, we tested for trends across ordered Agatston categories (600–999, 1000–1999, 2000–2999, > 3000) using the Cochran–Armitage test for: (1) patient-level accuracy (all patients), (2) sensitivity among ICA-positive patients, and (3) specificity among ICA-negative patients, each for both stenosis thresholds and for both readers. Inter-reader agreement for CCTA and ICA was quantified using Cohen’s kappa with 95% confidence intervals. Inter-reader agreement for CCTA and ICA was quantified using Cohen’s kappa with 95% confidence intervals. Kappa values were interpreted according to Landis and Koch [16]: < 0 poor, 0.00–0.20 slight, 0.21–0.40 fair, 0.41–0.60 moderate, 0.61–0.80 substantial, and 0.81–1.00 almost perfect agreement.

For vessel-level analyses, we used generalized estimating equations (GEE) with patient as the clustering unit (logit link, exchangeable correlation) to obtain population-averaged estimates with robust standard errors. We fitted separate models for accuracy (CCTA–ICA agreement), sensitivity (ICA-positive vessels), and specificity (ICA-negative vessels), including ordered Agatston category to test for monotonic trends (two-sided α = 0.05).

Statistical significance was defined as two-sided *p* < 0.05. All analyses were performed in R (version 4.4.0, The R Foundation).

## Results

### Patient population

**Fig. 1:**
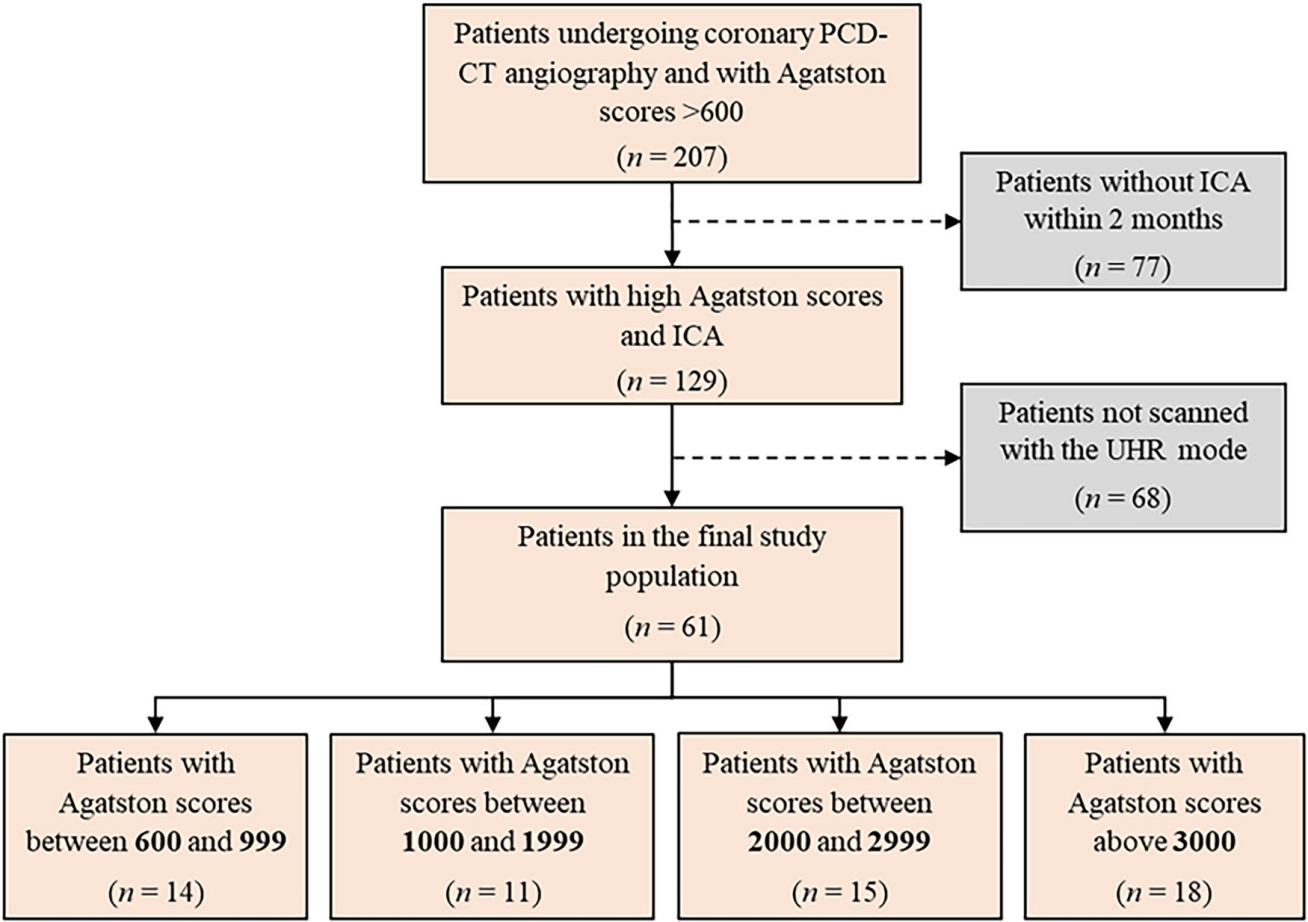
Study flowchart. CCTA, coronary CT angiography; ICA, invasive coronary angiography; PCD-CT, photon-counting detector CT; UHR, ultra-high resolution

From the 207 patients screened, 62 patients fulfilled the study inclusion criteria (Fig. 1). Fifty-eight (94%) patients underwent CCTA prior to transcatheter aortic valve replacement (TAVR). The median interval between CCTA and ICA was 2.5 days (IQR: 0–9). The mean age was 75 ± 8 years, and 26% (*n* = 16) were female. The average body mass index (BMI) was 26 ± 4 kg/m² (Table 1). The median Agatston score was 2423 (IQR: 1085–3340), and patients were categorized into four groups: 600–999 (*n* = 14), 1000–1999 (*n* = 14), 2000–2999 (*n* = 15), and > 3000 Agatston scores (*n* = 18).

**Table 1:** Patient characteristics

	Overall	Agatston score				
	(*n* = 61)	600–999 (*n* = 14)	1000–1999 (*n* = 14)	2000–2999 (*n* = 15)	> 3000 (*n* = 18)	*p*-value
Demographic and anthropometric						
Sex (female), *n* (%)	16 (26%)	5 (36%)	4 (29%)	4 (31%)	3 (19%)	0.66^a^
Age (years), mean ± SD	75 ± 8	70 ± 9	76 ± 9	74 ± 6	75 ± 8	0.226^b^
Weight (kg), mean ± SD	76 ± 15	75 ± 15	73 ± 14	70 ± 15	85 ± 14	0.28^b^
Height (cm), mean ± SD	170 ± 11	174 ± 10	169 ± 8	160 ± 10	178 ± 13	0.18^b^
BMI (kg/m^2^), mean ± SD	26 ± 4	24 ± 3	24 ± 4	24 ± 4	27 ± 6	0.93^b^
Blood pressure	126 ± 20/70 ± 14	130 ± 22/ 72 ± 13	129 ± 19 / 69 ± 14	123 ± 22/ 70 ± 11	126 ± 17 / 72 ± 17	0.86/0.41^b^
Medical history						
Hypertension, *n* (%)	45 (73%)	12 (85%)	10 (71%)	12 (80%)	11 (61%)	0.94^a^
Hyperlipidemia, *n* (%)	37 (61%)	5 (36%)	10 (71%)	11 (73%)	11 (61%)	0.34^a^
Diabetes, *n* (%)	25 (41%)	2 (14%)	8 (57%)	7 (54%)	8 (50%)	0.37^a^
Smoking, *n* (%)	21 (34%)	2 (14%)	8 (57%)	5 (38%)	6 (38%)	0.13^a^
Kidney disease, *n* (%)	10 (16%)	1 (7%)	2 (14%)	3 (20%)	4 (22%)	0.80^a^

^a^ Categorical: *p*-values from Fisher–Freeman–Halton test

^b^ Continuous: *p*-values from one-way ANOVA (normal distribution per Shapiro–Wilk) or Kruskal–Wallis when non-normally distributed

There were no differences between the Agatston score patient groups regarding age, sex, weight, height, BMI, and blood pressure during data acquisition (all *p* > 0.05). There was no significant correlation between Agatston scores and age, weight, height, and BMI, as well as the distribution of comorbidities (*p* > 0.05) (see Table 1).

### Stenosis distribution by Agatston score

According to ICA, the most common stenoses were found in the LAD, followed by the RCA and Cx artery (for further details see Supplementary Table 1). Patients with Agatston scores between 600 and 999 showed diameter stenoses above 50% and 70% in 9/56 (16%) and 4/56 (7%) of the segments, respectively. Patients with higher Agatston scores, particularly those above 3000, demonstrated a higher prevalence of coronary stenoses, primarily in the LAD and RCA.

### Diagnostic accuracy of CCTA and subgroup analysis by Agatston score

A vessel- and patient-based comparison between CCTA and ICA demonstrated consistently high diagnostic performance across Agatston score categories.

#### Patient-level analysis

**Fig. 2:**
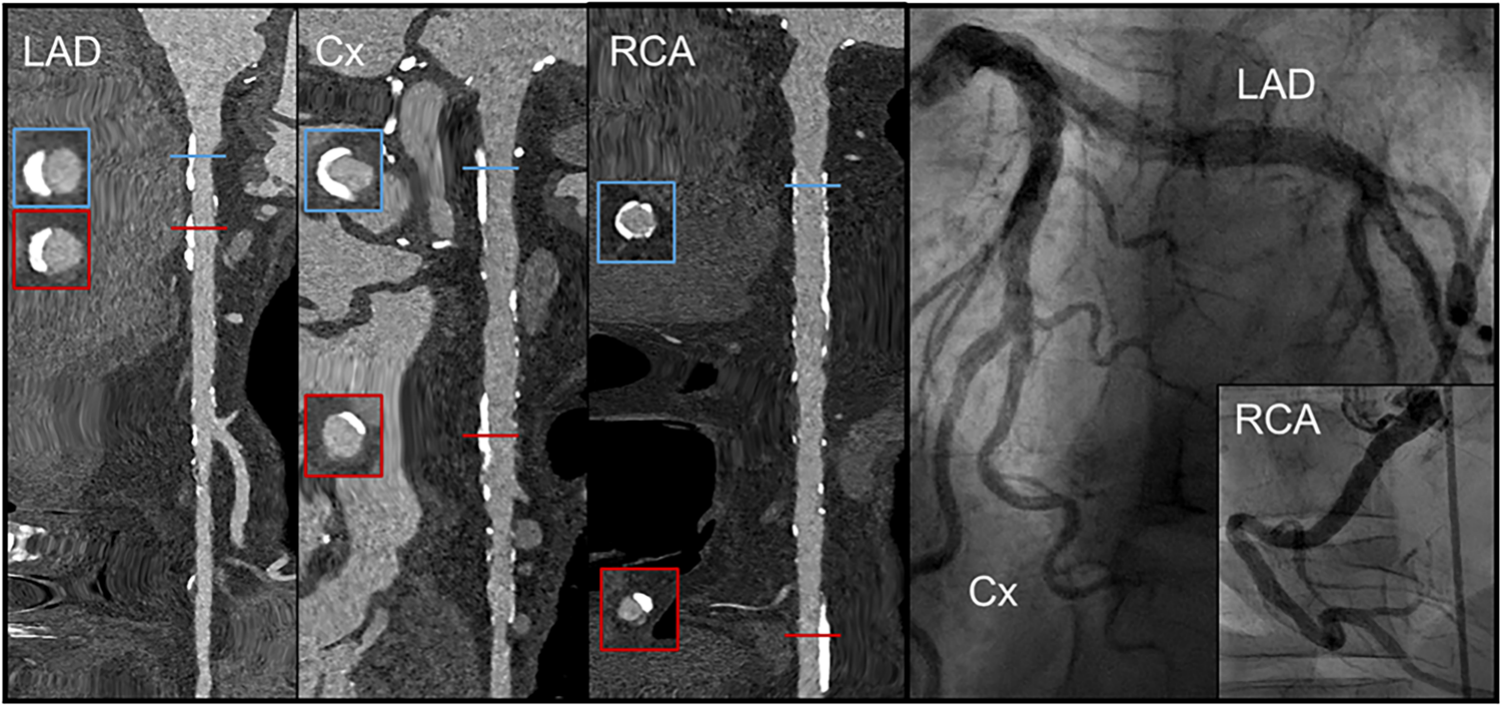
Ultra-high-resolution photon-counting detector coronary CT angiography and invasive coronary angiography in a 69-year-old male patient with severe aortic stenosis who was planned to undergo transcatheter aortic valve replacement. The Agatston score was 5221; CCTA and ICA showed no diameter stenosis > 50% in any major coronary artery. LAD, left anterior descending artery; Cx, circumflex artery; RCA, right coronary artery

**Fig. 3:**
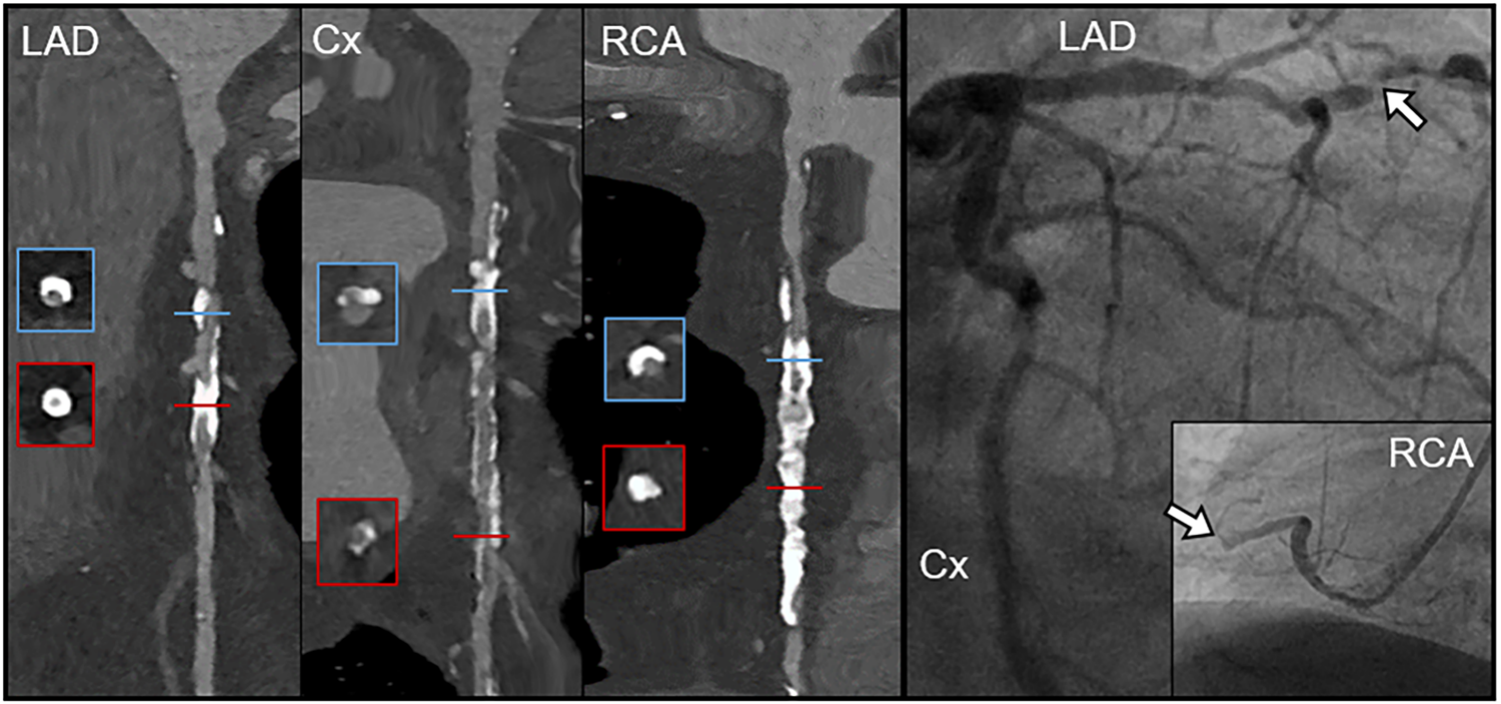
Ultra-high-resolution photon-counting detector coronary CT angiography and invasive coronary angiography in a 78-year-old male patient with severe aortic stenosis who was planned to undergo transcatheter aortic valve replacement. The Agatston score was 2871. Diameter stenosis > 70% in the LAD (left anterior descending artery) and RCA (right coronary artery) was found in CT and confirmed by ICA. LAD, left anterior descending artery; Cx, circumflex artery; RCA, right coronary artery

On a patient-based level, agreement for the 50% stenosis cut-off remained high in all groups, with overall sensitivity/specificity/accuracy of 98%/86%/94% (reader 1) and 90%/95%/92% (reader 2) and at a 70% stenosis cut-off 92%/100%/93% for reader 1 and 93%/94%/94% for reader 2 (Table 2 and Supplementary Fig. 1). Positive and negative predictive values are provided in Supplementary Table 2. Cochran–Armitage trend tests showed no evidence of decreasing accuracy, sensitivity, or specificity with increasing Agatston score category for both stenosis thresholds and for both readers (all *p* > 0.05). Representative image examples are provided in Figs. 2 and 3.

**Table 2:** Patient-based diagnostic performance of CCTA as compared to ICA

Patient-based		CCTA																Inter-reader agreement	
		50%-stenosis cut-off								70%-stenosis cut-off									
		Reader 1		Reader 2			Performance metrics			Reader 1		Reader 2						50% Cut-off	70% Cut-off
		0	1	0	1			Reader 1	Reader 2	0	1	0	1			Reader 1	Reader 2		
Overall																			
ICA	0	19	3	21	1	22	SE:	98% (94–100)	90% (83–99)	36	0	35	1	36	SE:	92% (80–96)	93% (85–99)	83% (69–97)	83% (69- 97)
	1	1	38	4	35	40	SP:	86% (76–93)	95% (89–100)	2	23	1	24	25	SP:	100% (100–100)	94% (87–99)		
		20	41	25	36	61	ACC:	94% (87–100)	92% (85–99)	38	23	36	25	61	ACC:	93% (88–100)	93% (86–100)		
600–999 AU																			
ICA	0	8	0	8	0	8	SE:	100% (100–100)	83% (62–100)	12	0	12	0	12	SE:	100% (100–100)	100% (100–100)	85% (57–100)	100% (100–100)
	1	0	6	1	5	6	SP:	100% (100–100)	100% (100–100)	0	2	0	2	2	SP:	100% (100–100)	100% (100–100)		
		8	6	9	5	14	ACC:	100% (100–100)	93% (74–100)	12	2	12	2	14	ACC:	100% (100–100)	100% (100–100)		
1000–1999 AU																			
ICA	0	4	1	5	0	5	SE:	100% (100–100)	90% (74–100)	6	0	6	0	6	SE:	91% (76–100)	88% (79–97)	71% (33–100)	100% (100–100)
	1	0	9	1	8	9	SP:	80% (52–98)	100% (100–100)	1	7	1	7	8	SP:	100% (100–100)	100% (100–100)		
		4	10	6	8	14	ACC:	93% (80–100)	93% (80–100)	7	7	7	7	14	ACC:	92% (80–100)	92% (80–100)		
2000–2999 AU																			
ICA	0	3	0	3	0	3	SE:	92% (77–100)	92% (85–100)	8	0	8	0	8	SE:	100% (100–100)	100% (100–100)	86% (57–100)	100% (100–100)
	1	1	11	1	11	12	SP:	100% (100–100)	100% (100–100)	0	7	0	7	7	SP:	100% (100–100)	100% (100–100)		
		4	11	4	11	15	ACC:	93% (80–100)	93% (80–100)	8	7	8	7	15	ACC:	100% (100–100)	100% (100–100)		
> 3000 AU																			
ICA	0	6	0	5	1	6	SE:	100% (100–100)	92% (80–100)	9	0	8	1	9	SE:	88% (72–100)	100% (100–100)	77% (46–100)	76% (46–100)
	1	0	12	1	11	12	SP:	100% (100–100)	83% (70–96)	1	8	0	9	9	SP:	100% (100–100)	89% (73–100)		
		6	12	6	12	18	ACC:	100% (100–100)	88% (72–100)	10	8	8	10	18	ACC:	94% (82–100)	94% (82–100)		

Inter-reader agreement between both readers on CCTA based on Cohen’s Kappa, 0 = no stenosis, 1 = stenosis, performance metrics with 95% confidence intervals in parentheses between ICA and CCTA for each reader

*ACC* accuracy, *AU* Agatston units, *CCTA* coronary computer tomography angiography, *ICA* invasive coronary angiography, *SE* sensitivity, *SP* specificity

#### Vessel-level analysis

On a vessel-based level, overall diagnostic performance remained consistently high, with accuracies of 94% (50% stenosis cut-off) and 95–96% (70% stenosis cut-off) for both readers (Supplementary Table 3 and Supplementary Fig. 2).

Across all categories, inter-reader agreement for CCTA remained high, with Cohen’s kappa coefficients between 0.71 and 1.00, corresponding to substantial to almost perfect agreement. After accounting for within-patient clustering using GEE, ordered Agatston score category was not associated with accuracy, sensitivity, or specificity for either stenosis threshold or reader (all *p* for trend > 0.05).

#### Discrepancy analysis

When analyzing discrepant versus concordant vessels, apart from sex and body weight, no significant associations were observed between baseline clinical characteristics and the presence of a discrepancy between CCTA and ICA. Technical acquisition parameters were not significantly different (Supplementary Table 4).

## Discussion

In patients with extensive coronary calcifications, noninvasive assessment of coronary artery disease (CAD) remains challenging. The diagnostic yield of conventional coronary CT angiography (CCTA) deteriorates in the presence of higher Agatston scores due to blooming artifacts obliterating the coronary lumen [5].

Our study specifically addressed this issue by evaluating the performance of the ultra-high resolution (UHR) mode of coronary photon-counting detector CT (PCD-CT) angiography in patients with a high coronary calcium burden (Agatston scores above 600). Our results indicate that coronary arteries can be reliably assessed using this CT scanner type and scan mode across all Agatston score groups, including those exceeding 3000, with neither sensitivity nor specificity showing a decline at high scores. We believe that this finding is clinically relevant as it supports the utility of PCD-CT as a filter test even in this challenging patient group.

The detector architecture of PCD-CT differs from that of conventional EID-CT systems. By directly converting incoming X-ray photons into electrical signals without intermediate light emission, PCD-CT reduces electronic noise and enhances spatial resolution [17]. The narrower detector pixels increase spatial resolution and reduce partial volume effects, thereby minimizing blooming around calcified plaques and preserving visibility of the vessel lumen. These technical improvements have been shown to reduce blooming artifacts from stents [18, 19] and also calcified plaques in initial PCD-CT studies [9, 11]. This may help to explain the consistent image quality seen in high Agatston score patients in our study.

The improved spatial resolution of PCD-CT also contributes to more accurate quantification of coronary stenoses. By reducing partial volume effects, PCD-CT enables reliable assessment of luminal narrowing, particularly in calcified segments [11]. Hagar et al previously demonstrated this benefit in patients with a median Agatston score of 414 [10]. Our study further adds to this evidence by showing that even with an extreme calcium burden with Agatston scores up to 7851 (median Agatston score 2423), the diagnostic performance of UHR PCD-CCTA remained high.

From a clinical perspective, these findings have important implications. In patients with high Agatston scores, UHR PCD-CCTA can reduce the need for unnecessary ICA, as previously suggested [6], thereby helping to avoid potential procedure-related complications associated with the invasive technique [20, 21]. This is particularly relevant in patients with advanced coronary or valvular atherosclerosis, where invasive procedures carry a risk for embolic events [22]. In addition, contrast-induced nephropathy (CIN) remains a concern, particularly when contrast media are administered intra-arterially. The guidelines of the European Society of Urogenital Radiology (ESUR) underscore this issue by highlighting the higher risk associated with intra-arterial (as opposed to intravenous) contrast administration for developing potential contrast-induced nephropathy [23], a result which was previously also demonstrated in controlled studies [24].

Most of our patients suffered from higher-grade aortic stenosis and were evaluated for TAVR [25]. Ruling out coronary stenosis as part of this CT examination, hereby avoiding subsequent ICA and its associated risks, simplifies care and potentially enhances patient safety. Thus, our study results support a patient-centered approach, in which CCTA serves as a first-line filter test to identify or exclude coronary stenosis in these patients.

In our subanalysis of discrepancies between CCTA and ICA, we found significant associations of discrepancies with sex and body weight, with female and higher weight being associated with a higher rate of discrepancy. Higher body weight may have negatively impacted image quality through the introduction of noise. The reason why female sex was associated with more discrepancy between modalities remains to be elucidated. The limited sample size in our study may have contributed to this statistical finding.

The following study limitations must be noted. First, this was a single-center study with a relatively small sample size, which may limit generalizability. As PCD-CT technology becomes more widely available, it will be crucial to validate these findings in multicenter prospective trials that include a larger patient population. In addition, the integration of quantitative plaque analysis and artificial intelligence-based tools could further augment diagnostic performance and reproducibility of the results [26]. Second, we did not include outcome data in our study to compare long-term outcomes between patients undergoing ICA vs. CCTA. Third, we did not evaluate whether PCD-CT might reduce the need for downstream testing as previously suggested [6]. Fourth, our cohort consisted predominantly of patients referred for TAVR evaluation, representing a highly selected population with advanced cardiac calcification. This selection may have affected diagnostic performance estimates, as patient compliance can often be limited in these patients. In a non-TAVR population, the diagnostic performance of UHR PCD-CCTA could potentially be higher. Fifth, we did not perform an a priori power calculation due to the exploratory nature and pragmatic Agatston strata. Subgroup sizes were modest, limiting the ability to exclude very small performance differences; however, formal trend tests across Agatston categories did not indicate declining accuracy or sensitivity. Finally, we did not perform dedicated analyses of subjective and objective image quality. As these metrics have been addressed in several prior PCD-CT studies [9, 10], we focused our work on the diagnostic performance of the modality.

In conclusion, our findings suggest that PCD-CT enables accurate evaluation of coronary arteries in patients with severe calcifications, with diagnostic performance maintained even at Agatston scores > 3000. By overcoming key limitations of EID-CCTA, this novel technology holds promise for improving noninvasive CAD assessment. Whether this may translate into a reduction of unnecessary invasive procedures remains to be shown in prospective comparative studies with patient outcome data.

## Supplementary information

ELECTRONIC SUPPLEMENTARY MATERIAL
