# Supplementary material for: Ultra-high resolution photon-counting detector coronary CT angiography: diagnostic accuracy in patients with high Agatston scores
Source: Eur Radiol. 2025 Nov 20;36(5):4017–25. doi: 10.1007/s00330-025-12158-z (PMC13086694; doi:10.1007/s00330-025-12158-z)
Supplement: Supplementary file 1 — ELECTRONIC SUPPLEMENTARY MATERIAL [file 330_2025_12158_MOESM1_ESM.pdf]

Ultra-High Resolution Photon-Counting Detector Coronary CT Angiography: Diagnostic Accuracy in Patients with High Agatston Scores

ELECTRONIC SUPPLEMENTARY MATERIAL

Supplemental Table 1. Vessel Characteristics.

	**LMCA**			**LAD**			**Cx**			**RCA**		
Stenosis	<50%	50-70%	>70%	<50%	50-70%	>70%	<50%	50-70%	>70%	<50%	50-70%	>70%
**ICA**												
Overall *(n=62)*:	60 (98%)	1 (2%)	0 (0%)	31 (51%)	12 (20%)	18 (29%)	43 (70%)	9 (15%)	9 (15%)	36 (59%)	10 (16%)	15 (25%)
AS: 600-999 *(n=14)*	14 (100%)	0 (0%)	0 (0%)	12 (86%)	2 (14%)	0 (0%)	12 (86%)	1 (7%)	1 (7%)	9 (64%)	3 (21%)	2 (14%)
AS: 1000-1999 *(n=14)*	14 (100%)	0 (0%)	0 (0%)	6 (46%)	3 (21%)	5 (33%)	11 (78%)	1 (8%)	2 (15%)	8 (57%)	4 (29%)	2 (14%)
AS: 2000-2999 *(n= 15)*	14 (93%)	1 (7%)	0 (0%)	4 (27%)	4 (27%)	7 (46%)	9 (60%)	5 (33%)	1 (7%)	9 (60%)	1 (7%)	5 (33%)
AS: >3000 *(n=18)*	18 (100%)	0 (0%)	0 (0%)	8 (45%)	4 (22%)	6 (33%)	11 (61%)	2 (11%)	5 (28%)	10 (56%)	2 (11%)	6 (33%)

AS= Agatston Score, CCTA = Coronary Computed Tomography Angiography, Cx = Left Circumflex Artery, ICA = Invasive Coronary Angiography, LAD = Left Anterior Descending Artery, LMCA = Left Main Coronary Artery, RCA = Right Coronary Artery

Supplemental Table 2. Patient-based positive and negative predictive values.

Agatston-score	50%- Cut-off				70% Cut-off			
	Reader 1		Reader 2		Reader 1		Reader 2	
	PPV	NPV	PPV	NPV	PPV	NPV	PPV	NPV
**Overall**	92.7%	95.0%	97.2%	84.0%	100.0%	94.7%	96.0%	97.2%
**600–999**	100.0%	100.0%	100.0%	88.9%	100.0%	100.0%	100.0%	100.0%
**1000–1999**	90.0%	100.0%	100.0%	83.3%	100.0%	85.7%	100.0%	85.7%
**2000–2999**	100.0%	75.0%	100.0%	75.0%	100.0%	100.0%	100.0%	100.0%
**>3000**	100.0%	100.0%	91.7%	83.3%	100.0%	90.0%	90.0%	100.0%

NPV= negative predictive value, PPV= positive predictive value

Supplemental Table 3. Vessel-based diagnostic performance of CCTA as compared to ICA.

Vessel -based		CCTA																Interreader Agreement	
		50%-Stenosis Cut-off								70%-Stenosis Cut-off									
		Reader 1		Reader 2			Performance Metrics			Reader 1		Reader 2			Performance Metrics			50%	70%
		0	1	0	1			Reader 1	Reader 2	0	1	0	1			Reader 1	Reader 2	Cut-off	Cut-off
**Overall**																			
ICA	0	161	9	163	8	171	SE:	91% (87-95)	89% (84-93)	197	7	197	7	204	SE:	90% (88-92)	93% (90-96)	88 % (81-95)	96 % (91-100)
	1	7	67	8	65	73	SP:	94% (91-97)	95% (92-98)	4	36	3	37	40	SP:	97% (95-99)	97% (94-100)		
		168	76	171	73	244	ACC:	94% (90-96)	94% (90-97)	201	43	200	44	244	ACC:	95% (92-98)	96% (93-99)		
**600-999 AU**																			
ICA	0	47	0	45	2	47	SE:	100% (100-100)	89% (81-97)	52	1	52	1	53	SE:	100% (100-100)	100% (100-100)	94 % (82-100)	100% (100-100)
	1	0	9	1	8	9	SP:	100% (100-100)	96% (90-100)	0	3	0	3	3	SP:	98% (95-100)	98% (95-100)		
		47	9	46	10	56	ACC:	100% (100-100)	95% (87-100)	52	4	52	4	56	ACC:	96% (93-100)	96% (93-100)		
**1000-1999 AU**																			
ICA	0	39	3	38	2	40	SE:	88% (81-96)	76% (64-86)	43	2	44	1	45	SE:	82% (70-90)	91% (82-92)	71 % (50-92)	95% (85-100)
	1	2	14	4	12	16	SP:	93% (85-99)	95% (89-100)	2	9	1	10	11	SP:	96% (91-100)	98% (91-100)		
		41	17	42	14	56	ACC:	92% (85-98)	90% (81-97)	45	11	45	11	56	ACC:	93% (86-100)	97% (89-100)		
**2000-2999 AU**																			
ICA	0	35	1	33	3	36	SE:	88% (79-96)	92% (84-98)	48	2	48	2	50	SE:	100% (100-100)	90% (81-97)	89% (77-100)	95% (85-100)
	1	3	21	2	22	24	SP:	97% (93-100)	92% (85-99)	0	10	1	9	10	SP:	96% (91-100)	96% (91-100)		
		38	22	35	25	60	ACC:	93% (86-100)	92% (85-99)	48	12	49	11	60	ACC:	96% (91-100)	95% (89-100)		
**>3000 AU**																			
IC	0	42	5	46	1	47	SE:	92% (85-98)	96% (90-100)	54	2	53	3	56	SE:	88% (77-93)	94% (88-100)	85% (72-98)	92% (81-100)

	1	2	23	1	24	25	SP:	89% (81-96)	98% (94-100)	2	14	1	15	16	SP:	96% (91-100)	95% (85-99)		
		44	28	47	2	72	ACC:	90% (82-97)	97% (92-100)	56	16	54	18	72	ACC:	94% (88-100)	94% (88-100)		

ACC= Accuracy, AU= Agatston Units, CCTA= Coronary Computer Tomography Angiography, ICA= Invasive Coronary Angiography, SE= Sensitivity, SP= Specificity, Interreader Agreement between both reader on CCTA based on Cohens Kappa, **Performance metrics with 95% confidence intervals in parentheses between ICA and CCTA for each reader, 0 = no Stenosis, 1 = Stenosis.**

Supplemental Table 4: Analysis of the discrepancy between CCTA and ICA.

Variable	no discrepancy (n=228)	discrepancy (n=16)	*p*-value
Sex (female)	52 (23%)	8 (50%)	<0.05[1]
Hyperlipidemia	85 (37%)	5 (31%)	0.32[1]
Kidney disease	28 (12%)	2 (12%)	0.43[1]
Hypertension	160 (70%)	12 (75%)	0.46[1]
Smoking	72 (32%9	5 (31%)	0.53[1]
Diabetes	94 (41%)	7 (44%)	0.84[1]
Weight (kg)	74± 16	77± 23	<0.05[2]
Height (cm)	170±14	172± 19	0.22[2]
Body mass index	23±5	25±4	0.08[2]
Systolic BP (mmHg)	139±19	126±35	0.29[2]
Diastolic BP (mmHg)	73±12	71±19	0.61[2]
Tube voltage (kVp)	120+20	120+20	0.46[2]
Tube current (mAs)	38±15	49±19	0.08[2]
$CTDI_{vol}$ (mGy)	41±16	43±19	0.72[2]
DLP (mGy·cm)	627±340	702±336	0.72[2]
Heart rate (bpm)	75±17	75±17	0.33[2]

Supplemental Table 4: Comparison of baseline characteristics of vessels with (n= 16) and without (n= 228) discrepancy between ICA and CCTA, with corresponding p-values.

¹ Categorical: *p*-values from Fisher–Freeman–Halton test, numbers in total (percentage n=244); ² Continuous: *p*-values from one-way ANOVA (normal per Shapiro–Wilk) or Kruskal–Wallis when non-normal, numbers in Median (IQR)

BP = Blood pressure, *$CTDI_{vol}$* = Volume computed tomography dose index, *DLP* = Dose length product, *bpm* = beats per minute

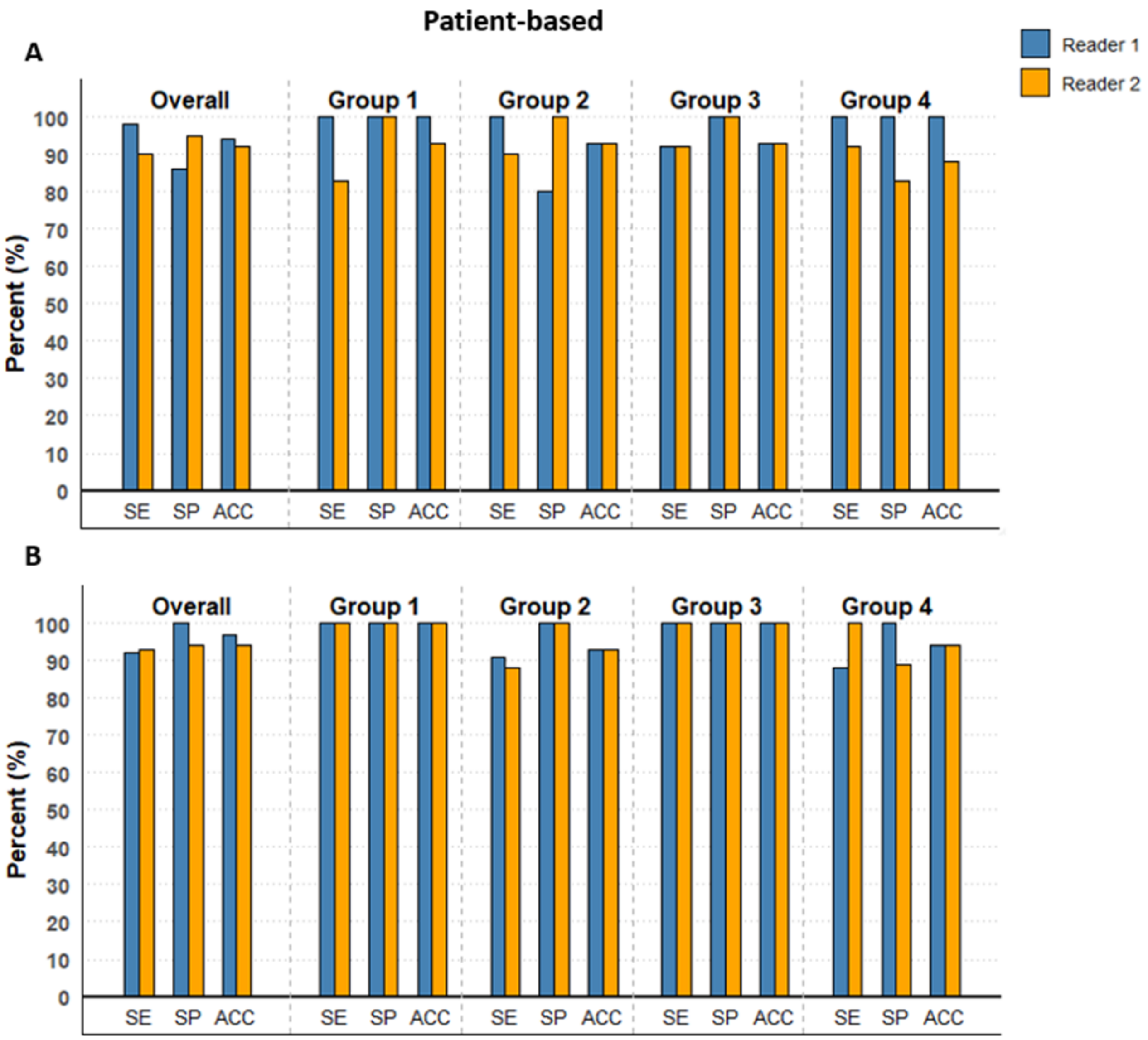

Supplemental Figure 1: Patient-based comparison between Invasive Coronary Angiography and Coronary CT Angiography. Group 1: 600–999 (n = 14), Group 2: 1000–1999 (n = 14), Group 3. 2000–2999 (n = 15), and group 4: >3000 (n = 18) *A: >50% diameter stenosis, B: >70% diameter stenosis, ACC= accuracy, SE= sensitivity, SP= specificity*

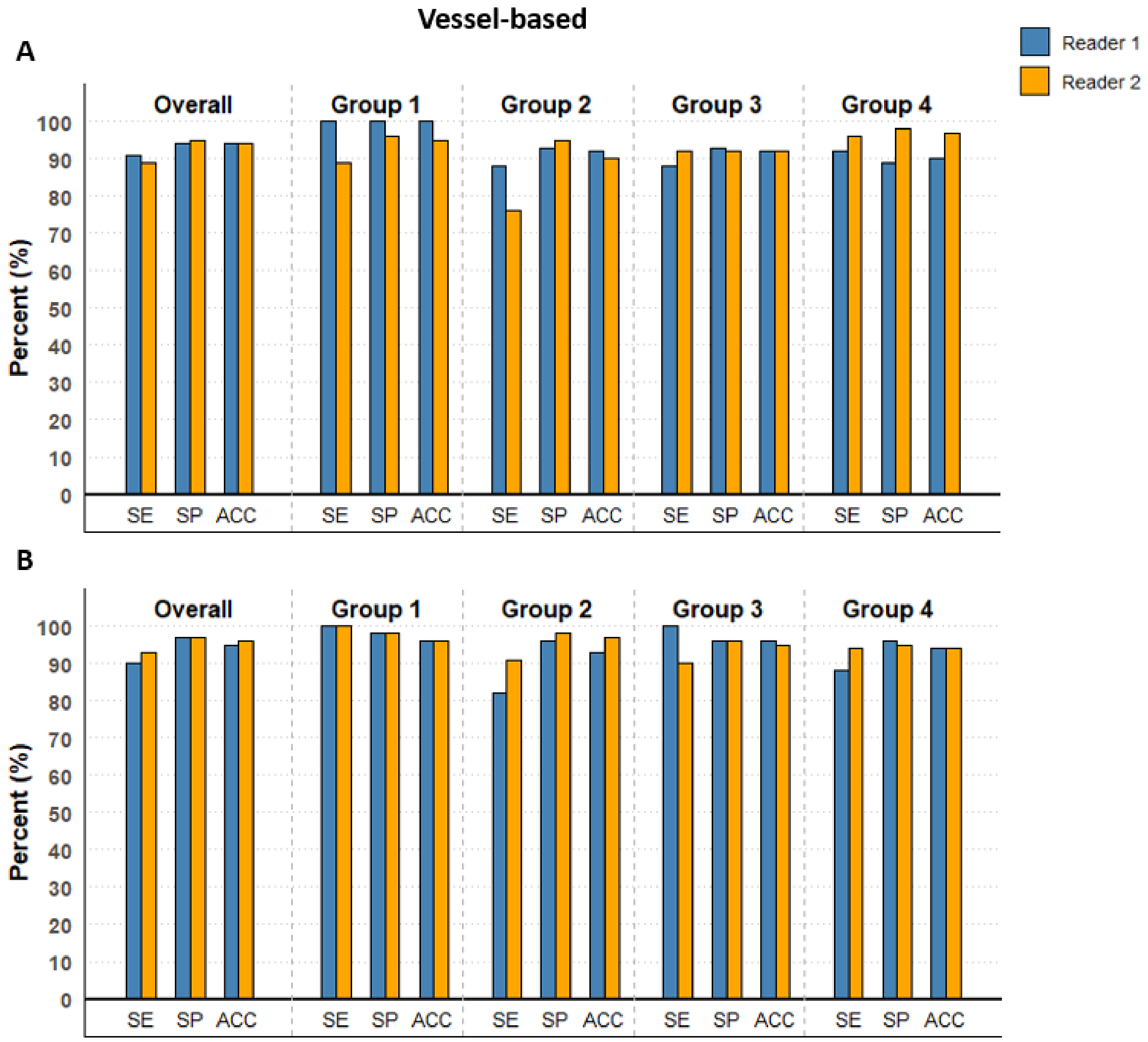

Supplemental Figure 2: Vessel-based comparison between Invasive Coronary Angiography and Coronary CT Angiography. Group 1: 600–999 (n = 14), Group 2: 1000–1999 (n = 14), Group 3. 2000–2999 (n = 15), and group 4: >3000 (n = 18)

A: >50% diameter stenosis, B: >70% diameter stenosis, ACC= accuracy, SE= sensitivity, SP= specificity
